# Supplementary material for: Ethanol binge drinking exposure affects alveolar bone quality and aggravates bone loss in experimentally-induced periodontitis
Source: PLoS One. 2020 Jul 30;15(7):e0236161. doi: 10.1371/journal.pone.0236161 (PMC7392256; doi:10.1371/journal.pone.0236161)
Supplement: S1 File — (DOCX) [file pone.0236161.s001.docx]

EtOH and periodontitis: descriptive analyses.

| Tb.Th | | | | |
| --- | --- | --- | --- | --- |
| Groups | Control | EtOH | Perio | EtOH + perio |
|  |  |  |  |  |
| Minimum | 0.1297 | 0.1014 | 0.07710 | 0.07006 |
| 25% percentile | 0.1297 | 0.1053 | 0.08116 | 0.07100 |
| Median | 0.1581 | 0.1289 | 0.1012 | 0.07608 |
| 75% percentile | 0.1752 | 0.1500 | 0.1144 | 0.08979 |
| Maximum | 0.1927 | 0.1532 | 0.1162 | 0.09627 |
|  |  |  |  |  |
| Mean | 0.1571 | 0.1286 | 0.09962 | 0.07953 |
| Std. Deviation | 0.02399 | 0.02246 | 0.01590 | 0.01065 |
| Std. Error of mean | 0.009066 | 0.007940 | 0.006011 | 0.004763 |

| Tb.N | | | | |
| --- | --- | --- | --- | --- |
| Groups | Control | EtOH | Perio | EtOH + perio |
|  |  |  |  |  |
| Minimum | 0.3847 | 0.2928 | 0.2170 | 0.2105 |
| 25% percentile | 0.4113 | 0.3228 | 0.2262 | 0.2106 |
| Median | 0.4352 | 0.3595 | 0.2405 | 0.2175 |
| 75% percentile | 0.4787 | 0.4148 | 0.2832 | 0.2639 |
| Maximum | 0.4986 | 0.4155 | 0.2973 | 0.2772 |
|  |  |  |  |  |
| Mean | 0.4408 | 0.3617 | 0.2510 | 0.2307 |
| Std. Deviation | 0.03865 | 0.04722 | 0.03072 | 0.03163 |
| Std. Error of mean | 0.01461 | 0.01785 | 0.01086 | 0.01582 |

| BV/TV% | | | | |
| --- | --- | --- | --- | --- |
| Groups | Control | EtOH | Perio | EtOH + perio |
|  |  |  |  |  |
| Minimum | 0.2119 | 0.1101 | 0.2053 | 0.1253 |
| 25% percentile | 0.2185 | 0.1432 | 0.2068 | 0.1579 |
| Median | 0.2414 | 0.1833 | 0.2459 | 0.1929 |
| 75% percentile | 0.2820 | 0.2516 | 0.2568 | 0.2061 |
| Maximum | 0.3474 | 0.2531 | 0.2611 | 0.2187 |
|  |  |  |  |  |
| Mean | 0.2537 | 0.1898 | 0.2379 | 0.1842 |
| Std. Deviation | 0.04570 | 0.05781 | 0.02278 | 0.03488 |
| Std. Error of mean | 0.01616 | 0.02044 | 0.008611 | 0.01560 |

| Alveolar Bone Loss | | | | |
| --- | --- | --- | --- | --- |
| Groups | Control | EtOH | Perio | EtOH + perio |
|  |  |  |  |  |
| Minimum | 0.7117 | 0.7167 | 0.8650 | 0.9500 |
| 25% percentile | 0.7367 | 0.7567 | 0.8862 | 0.9604 |
| Median | 0.7717 | 0.8383 | 0.9338 | 1.059 |
| 75% percentile | 0.8042 | 0.8667 | 0.9558 | 1.148 |
| Maximum | 0.8483 | 0.9783 | 0.9583 | 1.155 |
|  |  |  |  |  |
| Mean | 0.7742 | 0.8248 | 0.9232 | 1.056 |
| Std. Deviation | 0.04393 | 0.08797 | 0.03860 | 0.1003 |
| Std. Error of mean | 0.01553 | 0.03325 | 0.01576 | 0.05014 |

| Exposed Root Area | | | | |
| --- | --- | --- | --- | --- |
| Groups | Control | EtOH | Perio | EtOH + perio |
|  |  |  |  |  |
| Minimum | 1.686 | 1.766 | 2.001 | 2.399 |
| 25% percentile | 1.697 | 1.777 | 2.248 | 2.487 |
| Median | 2.028 | 2.106 | 2.419 | 2.723 |
| 75% percentile | 2.086 | 2.248 | 2.616 | 3.352 |
| Maximum | 2.168 | 2.401 | 2.851 | 3.605 |
|  |  |  |  |  |
| Mean | 1.945 | 2.054 | 2.428 | 2.880 |
| Std. Deviation | 0.2014 | 0.2531 | 0.2675 | 0.4804 |
| Std. Error of mean | 0.08222 | 0.08948 | 0.1011 | 0.2149 |

| **Bodyweight evaluation** | | | | | | | | | | | | |
| --- | --- | --- | --- | --- | --- | --- | --- | --- | --- | --- | --- | --- |
| Groups | Control | | | EtOH | | | Periodontitis | | | EtOH + Periodontitis | | |
|  | Mean | SEM | N | Mean | SEM | N | Mean | SEM | N | Mean | SEM | N |
| Day 0 | 223.37 | 4.574532 | 8 | 217.75 | 5.700094 | 8 | 230.5 | 5.861497 | 8 | 241.25 | 6.906906 | 8 |
| Day 7 | 242.75 | 5.999256 | 8 | 229.75 | 6.656763 | 8 | 238.25 | 5.924375 | 8 | 251.57 | 9.115286 | 7 |
| Day 14 | 254.25 | 5.157346 | 8 | 233.12 | 6.908684 | 8 | 239 | 5.982116 | 8 | 258.8 | 8.811356 | 5 |
| Day 21 | 251.12 | 5.309821 | 8 | 234.75 | 6.912075 | 8 | 240.62 | 7.606242 | 8 | 259.2 | 10.2098 | 5 |
| Day 28 | 259.25 | 4.685654 | 8 | 237.62 | 7.768842 | 8 | 241.87 | 7.670532 | 8 | 263.2 | 10.62732 | 5 |
